# Alternative functional *in vitro* models of human intestinal epithelia

**DOI:** 10.3389/fphar.2013.00079

**Published:** 2013-07-08

**Authors:** Amanda L. Kauffman, Alexandra V. Gyurdieva, John R. Mabus, Chrissa Ferguson, Zhengyin Yan, Pamela J. Hornby

**Affiliations:** ^1^Biologics Research, Biotechnology Center of Excellence, Janssen Pharmaceutical Companies of Johnson & JohnsonSpring House, PA, USA; ^2^Drug Discovery, Analytical Science, Janssen Pharmaceutical Companies of Johnson & JohnsonSpring House, PA, USA

**Keywords:** human intestinal epithelial cell (hInEpC), induced pluripotent stem cell (iPSC), permeability, Transepithelial Electrical Resistance (TEER), neonatal Fc receptor (FcRn)

## Abstract

Physiologically relevant sources of absorptive intestinal epithelial cells are crucial for human drug transport studies. Human adenocarcinoma-derived intestinal cell lines, such as Caco-2, offer conveniences of easy culture maintenance and scalability, but do not fully recapitulate *in vivo* intestinal phenotypes. Additional sources of renewable physiologically relevant human intestinal cells would provide a much needed tool for drug discovery and intestinal physiology. We compared two alternative sources of human intestinal cells, commercially available primary human intestinal epithelial cells (hInEpCs) and induced pluripotent stem cell (iPSC)-derived intestinal cells to Caco-2, for use in *in vitro* transwell monolayer intestinal transport assays. To achieve this for iPSC-derived cells, intestinal organogenesis was adapted to transwell differentiation. Intestinal cells were assessed by marker expression through immunocytochemical and mRNA expression analyses, monolayer integrity through Transepithelial Electrical Resistance (TEER) measurements and molecule permeability, and functionality by taking advantage the well-characterized intestinal transport mechanisms. In most cases, marker expression for primary hInEpCs and iPSC-derived cells appeared to be as good as or better than Caco-2. Furthermore, transwell monolayers exhibited high TEER with low permeability. Primary hInEpCs showed molecule efflux indicative of P-glycoprotein (Pgp) transport. Primary hInEpCs and iPSC-derived cells also showed neonatal Fc receptor-dependent binding of immunoglobulin G variants. Primary hInEpCs and iPSC-derived intestinal cells exhibit expected marker expression and demonstrate basic functional monolayer formation, similar to or better than Caco-2. These cells could offer an alternative source of human intestinal cells for understanding normal intestinal epithelial physiology and drug transport.

## Introduction

The use of *in vitro* cell models for human drug transport studies has focused on intestinal epithelial cells, as these cultures contain primarily absorptive cells. While isolated human intestinal epithelial cells (hInEpCs) retain important *in vivo* anatomical and biochemical features, they are difficult to culture and have limited viability. As a result, immortalized human adenocarcinoma cell lines have been extensively used to study absorption mechanisms. While immortalized cells offer many advantages, extrapolation of data generated with these cell lines to *in vivo* conditions is often difficult, as these cells originated from tumors and are therefore not representative of the true physiological environment (Le Ferrec et al., 2001). In addition, these cells form monolayers that are widely used for small molecule intestinal permeation *in vitro* studies (below). But, with increasing numbers of biotechnology protein therapeutics and novel scaffolds available, which open the possibility for oral delivery, there is a need for alternatives that more closely recapitulate the physiology of the intestinal epithelial cell.

The human colorectal adenocarcinoma cell line Caco-2 is frequently used for drug absorption studies, particularly in the context of small molecules (Le Ferrec et al., 2001; Balimane and Chong, 2005). Caco-2 cells are easy to culture and have the capacity to spontaneously differentiate into cells possessing the morphology and function of enterocytes, the absorptive cells of the intestine (Balimane and Chong, 2005). Caco-2 cells are commonly cultured on semi-permeable inserts in a transwell format, where the cells form a polarized monolayer (Leonard et al., 2000; Le Ferrec et al., 2001), and the transport of molecules between the apical and basolateral chambers can be easily evaluated. While Caco-2 cells are a good model for observation of passive transcellular and paracellular permeability (Balimane and Chong, 2005), there are differences in cytokine production and cytokine receptor expression between Caco-2 cells and normal epithelial cells (Aldhous et al., 2001). In addition, Caco-2 cells under-express transporters and metabolizing enzymes relative to *in vivo* tissue, potentially excluding mechanisms crucial for drug absorption studies (Balimane and Chong, 2005).

Due to the limitations of immortalized intestinal cell lines, many studies have focused on the use of primary hInEpCs as a more physiologically relevant cell-based model (Perreault and Beaulieu, 1998; Aldhous et al., 2001; Ootani et al., 2009; Lahar et al., 2011). However, stocks of these cells are difficult to maintain due to limited donors and low viability in culture. Recently, commercial sources of primary hInEpCs were made available (Lonza; Walkersville, MD), which greatly increase the convenience of obtaining primary cell stocks. Commercial quality control data suggest that these primary hInEpCs have the capacity to form monolayers with tight junctions and express general epithelial markers, such as cytokeratins 8 and 18 (Bosch et al., 1988); however, little characterization has be done on their expression of intestinal cell type-specific markers or transport function. Other efforts to enable long-term culture of primary cells and enhance physiological conditions have led to the development of 3-dimensional (3D) models of the intestinal epithelium, which have focused on the use of primary intestinal stem cells and directed differentiation of pluripotent stem cells.

Stem cells have the capacity to self-renew and differentiate into the various cell lineages that make up specific tissue types. For example, intestinal stem cells are responsible for the self-renewal of the gut epithelium, and have been used in developing 3D intestinal models. Leucine-rich repeat-containing G protein-coupled receptor 5 (LGR-5)-positive stem cells can be isolated from primary intestinal tissue and grown as 3D intestinal organoids with crypt-villius physiology and culturing capacity up to 8 months (Sato et al., 2009). While 3D organoids derived from primary intestinal stem cells appear to possess physiologically relevant phenotypes, they cannot be used to assess classical functionality typically determined within 2-dimensional transwell cultures, such as the formation of monolayers with tight junctions and intestinal permeability and transport.

An additional source of human intestinal cells is possible through directed differentiation of pluripotent stem cells to intestinal cell lineages. The recent advent of human induced pluripotent stem cells (iPSCs) has provided a huge therapeutic potential as a tool for drug discovery, as patient-specific somatic cells can be reprogrammed into an embryonic stem cell-like state that can be directly differentiated to a specific cell type of interest for more physiologically relevant disease modeling. Induced human intestinal organoids (iHIOs) have recently been derived from iPSCs (Spence et al., 2010), which are capable of expressing epithelial and intestinal markers such as caudal type homeobox 2 (CDX2) (hindgut marker), E-Cadherin (cell-to-cell junction marker), and Villin (epithelial brush border marker). However, to our knowledge, the methods for differentiating these into a polarized epithelial monolayer similar to Caco-2 have not been reported.

**Figure 1:**
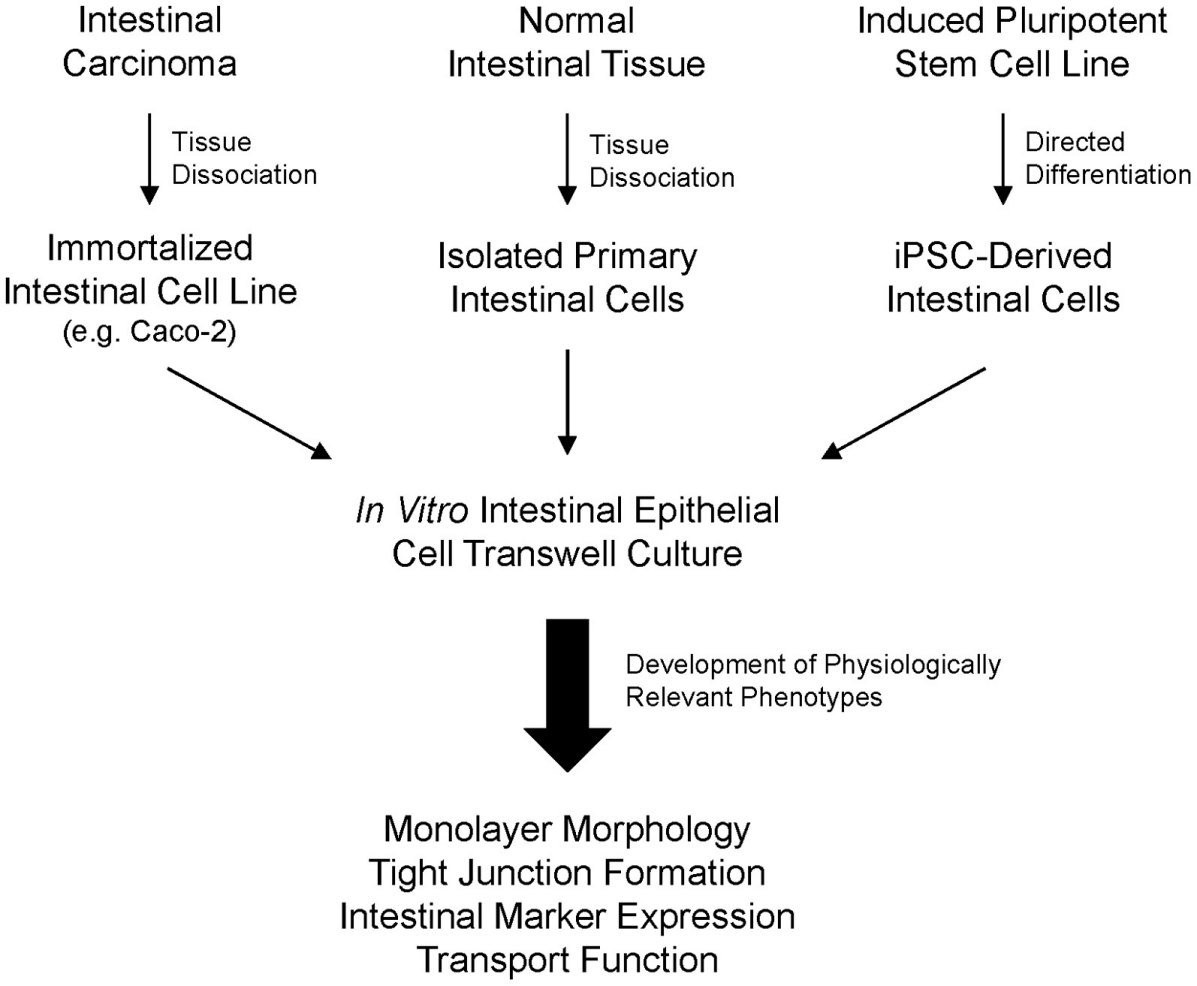
**Overview of isolation and utilization of human intestinal epithelial cells in transwell cultures for *in vitro* assays.** Major sources of human intestinal cells include intestinal carcinoma tissue, normal intestinal tissue, or directed differentiation of induced pluripotent stem cells.

In this study, we assessed expression of markers and functional activity of the newly commercially available primary hInEpCs and iPSC-derived intestinal cells compared to Caco-2 (Figure 1) in cell-based *in vitro* assays. We adapted our previously described 3D intestinal organogenesis to differentiation within transwells. Intestinal marker expression, formation of monolayers with tight junction formation and functional molecule transport and binding were evaluated.

## Materials and methods

### Cell culture

#### Intestinal cells

Human primary small intestinal epithelial cells (Lonza; Walkersville, MD) from 3 donors (Donor A: Lot # 0000258132; Donor B: Lot # 0000256741; Donor C: Lot # 0000256744) were thawed and cultured in transwell inserts for 10–11 days in SmGM-2 media (Lonza), according to manufacturer's instructions. Caco-2 colorectal adenocarinoma-derived cells (ATCC; Manassas, VA) were cultured in transwell inserts for 14–21 days in Caco-2 media (10% fetal bovine serum, 1X non-essential amino acids, 1X sodium pyruvate, and 6 mM L-Glutamine in Dulbecco's Modified Eagle's Medium (DMEM) High Glucose, reagents from Life Technologies; Carlsbad, CA).

#### iPSCs

A1145A and B2198A (Johnson & Johnson; Spring House, PA) were produced from human kidney-derived cells using retroviral (Takahashi et al., 2007) and modified mRNA (non-viral) reprogramming methods (Yakubov et al., 2010), respectively. C2198A and C2200B (Johnson & Johnson; Spring House, PA) were produced from human umbilical tissue-derived cells by modified RNA reprogramming methods. D2043A (System Biosciences; Mountain View, CA) was derived from human foreskin fibroblasts by retroviral methods. iPSC lines were maintained in mTeSR1 culture media (STEMCELL Technologies; Vancouver, BC) on culture dishes/flasks coated with Geltrex (Life Technologies) and passaged by Dispase (STEMCELL Technologies) dissociation every 3–5 days as previously described (McCracken et al., 2011).

### iPSC directed differentiation

#### Transwell differentiation

Semi-permeable transwell inserts were coated apically with Geltrex (Life Technologies) prior to plating iPSCs within 12-well transwell tissue culture plates. iPSCs were plated apically as cell clumps (5–10 cells per clump) at a density of 3000–6000 clumps per transwell insert. Two to three days after plating, iPSCs were differentiated within transwell inserts modified previously reported methods (Spence et al., 2010) into definitive endoderm using GDF8 with GSK3b inhibitor and B27 supplement then differentiated to hindgut using Keratinocyte Growth Factor and Retinoic Acid then EGF, Noggin, and R-Spondin1 for >26 days (Kauffman et al., Submitted) on both sides of the transwell insert; however, the many spheroid structures observed at Stage 2 were maintained as part of the adherent layer throughout Stage 3 differentiation (Table 1) for up to 31 days.

**Table 1:** **Summary of growth factors and differentiation cell culture conditions used for transwell differentiation, relative to previously established intestinal organogenesis to 3D organoids**.

**Stage**	**Growth factors**	**Days**	**Cell type**	**Stage markers**	**Organoid culture**	**Transwell culture**
1	GDF-8, GSKi, B27	3	Definitive endoderm	SOX17, CXCR4	2D Standard tissue culture plate	2D Transwell
2	KGF, RA	7	Hindgut	CDX2	2D Excise spheroids from standard plate	2D Transwell (No Spheroid Excision)
3	EGF, Noggin, R-Spondin1	26+	Intestinal	E-Cad, CDX2, Villin	3D Intestinal matrigel	2D Transwell

### Monolayer assessment

#### Transepithelial electrical resistance (TEER)

TEER of transwell cultures was recorded in measurements of Ohms using an Epithelial Volt Ohm Meter (EVOM)^2^ and electrode set (World Precision Instruments; Sarasota, FL). Raw data was converted to Ω × cm^2^ based on area of transwell plate inserts (1.12 cm^2^).

#### FITC-dextran permeability

Cells in 24-well transwell plates were washed twice with DPBS (Life Technologies). To the apical side, 200 μ L of 12.5 mg/mL Fluorescein isothiocyanate–dextran, molecular weight 150 kDa (FD150) (Sigma–Aldrich) diluted in Caco-2 media (*pH* = 6.0) was. To the basolateral side, 500 uL of Caco-2 media (*pH* = 7.4) was added. After a 90-min incubation at 37°C, 100 uL of media was collected from the basolateral chamber and analyzed for the presence of FITC-Dextran using a SpectraMax M5 microplate reader (Molecular Devices; Sunnyvale, CA).

### P-glycoprotein transport assay

Primary hInEpCs were dosed on the apical or basolateral side with a 2 mM mix of Digoxin and Atenolol in HBSSg [2 mM glucose and 10 mM 4-(2-hydroxyethyl)-1-piperazineethanesulfonic acid in Hank's Balanced Salt Solution (HBSS)] with calcium and magnesium, *pH* = 7.4) and incubated for 90 min at 37°C in the presence or absence of 10 μM Cyclosporin A (CSA). Samples were collected from both the apical/donor and basolateral/receiver chambers and analyzed by LCMS. Digoxin and Atenolol levels in each condition were used to calculate apparent permeability (*P*app = δ*Cr*/δ*t*) × *Vr* / (*A* × *C*_0_)) in the apical to basolateral (A–B) or basolateral to apical (B–A) direction. δCr = final receiver concentration; δt = assay time; Vr = receiver volume; A = transwell growth area; C_0_ = initial apical concentration. To ensure monolayer integrity during the assay, all wells were dosed apically with 100 μ g/mL Lucifer Yellow (LY) at the start of Digoxin and Atenolol incubation, and samples were collected from the basolateral chamber for analysis by SpectraMax M5 microplate reader at the end of the 90 min incubation. Only transwells with a LY Papp(A–B) of < 1 × 10^−6^ cm/s were used in calculating final Papp Ratios for Digoxin and Atenolol. Based on this cutoff, one of 8 wells each for hInEpC Donors A and B, and two of 8 wells for Donor C was excluded from data analysis.

### mRNA expression

RNA was harvested from Caco-2, primary hInEpCs, or iPSCs before or after differentiation to Stage 3 by RNeasy Mini kit (Qiagen; Germantown, MD), and reverse transcribed to cDNA using the RT^2^ First Strand kit (Qiagen). Using 30 ng/μ L starting cDNA, samples were used in reactions within Custom RT^2^ Profiler PCR array containing probes supplied by the manufacturer (SABiosciences; Valencia, CA) for intestinal and control markers (Table 2), following reaction cycling conditions outlined in the manufacturer's protocol. Data analysis was performed using the Δ CT method, where raw *Ct* values were normalized to housekeeping gene 60S acidic ribosomal protein P0 (RPLP0) before comparing expression relative to Caco-2. Expression levels for primary hInEpCs represents the average across all three donors used in this study.

**Table 2:** **List of probes used in Custom RT^2^ Profiler PCR array mRNA expression analyses of intestinal and control markers in intestinal epithelial cells**.

**Gene**	**NCBI reference no**	**SAB catalog no**
E-Cadherin	NM_004360	PPH00135
CDX2	NM_001265	PPH13618
KLF5	NM_001730	PPH00434
Villin	NM_007127	PPH23365
SOX9	NM_000346	PPH02125
LGR5	NM_003667	PPH13346
ASCL2	NM_005170	PPH12852
MUC2	NM_002457	PPH06990
Chromogranin A	NM_001275	PPH01181
LYZ	NM_000239	PPH14748
VIM	NM_003380	PPH00417
FcRn	NM_004107	PPH11194
CXCR4	NM_003467	PPH00621
PDX1	NM_000209	PPH05536
OCT4	NM_002701	PPH02394
TNNT2	NM_000364	PPH02619
PAX6	NM_000280	PPH02598
TUBB3	NM_006086	PPH02607
RPLP0	NM_001002	PPH21138

*Probes for the NCBI Reference sequence numbers listed were obtained from SABiosciences (SAB) using the catalog numbers provided*.

### Protein expression

#### Flow cytometry

**Figure A1:**
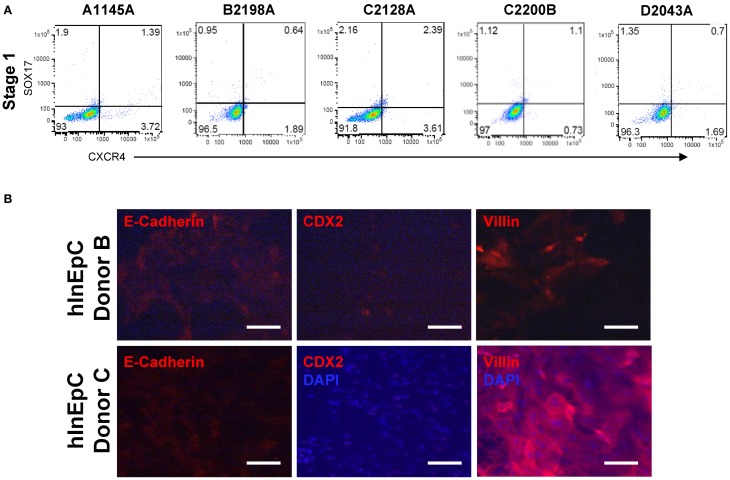
**Additional protein expression data. (A)** Isotype controls used for flow cytometry plot gating in Figure 3B. **(B)** Expression of intestinal markers E-Cadherin, CDX2, and Villin is shown in primary hInEpC Donors B (top row) and C (bottom row).

Undifferentiated or Stage 1 iPSCs were detached by treatment with Accutase (Sigma-Aldrich; St. Louis, MO) and stained for viability by Near Infrared Live/Dead kit (Invitrogen; Carlsbad, CA) before fixation in Cytofix Buffer (BD Biosciences; San Jose, CA). For pluripotent marker analyses, cells were stained using Human Pluripotent Stem Cell Transcription Factor Analysis or Human Pluripotent Stem Cell Sorting and Analysis kits (BD Biosciences). For definitive endoderm marker analysis, cells were stained with a 1:5 dilution of PE-conjugated mouse anti-human CD184/ C-X-C chemokine receptor type 4 (CXCR4) (306506, Biolegend; San Diego, CA) before permeabilized with Phosflow Perm Buffer (BD Biosciences) and stained with a 1:5 dilution of APC-conjugated goat polyclonal anti-human Sry-related HMG box 17 (SOX17) (IC1924A, R&D Systems; Minneapolis, MN). For analysis of surface expression of neonatal Fc receptor (FcRn), Caco-2 and primary hInEpCs were dissociated from transwell culture by Accutase, incubated with 50 μg/mL affinity purified rat anti-human FcRn polyclonal antibody (generated in house), followed by incubation with 7.5 μg/mL FITC Donkey anti-rat IgG (FAb2) secondary antibody (109-006-006, Jackson Immunoresearch; West Grove, PA). Fluorescence of stained cells was measured in conjunction with appropriate compensation controls (BD Biosciences) by flow cytometry using an LSR Fortessa FACS Sorter (BD Biosciences). Raw data was analyzed by FlowJo analysis software (Tree Star; Ashland, OR), with gating parameters set based on isotype controls (Figure A1A).

#### Immunofluorescence

iPSC-derived cells Stage 2 iPSC-derived cells were fixed in 4% paraformaldehyde (Electron Microscoopy Sciences; Hatfield, PA) before permeabilization with 0.5% Triton X-100 (Electron Microscoopy Sciences). After addition of Image-iT® FX signal enhancer (Invitrogen), cells were treated with 1X Blocking Buffer (Sigma) before exposure to a 1:50 dilution of E-Cadherin, CDX2, or Villin antibodies (Dako), using manufacturer recommended concentrations. Immunoreactivity to primary antibodies was detected with a 1:500 dilution of AF568-conjucated goat anti-mouse antibody (Invitrogen). Prolong GOLD antifade with DAPI (Invitrogen) was added to wells prior to visualizing on a Nikon SMZ-1500 fluorescence dissecting microscope.

### FcRn-dependent immunoglobulin G (IgG) binding

Caco-2, hInEpCs (Donor A), or iPSC-derived cells were detached from transwells by Accutase (Sigma) treatment and washed twice with Dulbecco's Phosphate-Buffered Saline (DPBS) (Life Technologies). Cells were transferred in DPBS to MesoScale Discovery (MSD) High Bind plates (MSD; Rockville, MD) at a density of 1 × 10^4^ or 2.5 × 10^4^ cells per well, and incubated at room temperature for 2 h to allow attachment to the plate surface. Plates were then blocked with 20% Fetal Bovine Serum (Life Technologies) and 0.18% Sodium Azide (VWR International; Radnor, PA) for 15 min at room temperature. Wells were washed once with DPBS at *pH* 6.0 before incubation with FcRn-binding variants (anti-RSV N434A or anti-RSV H435A in DPBS at *pH* 6.0) for 90 min at 37°C. Plates were washed 3 times with DPBS at *pH* 6.0, and cells were incubated with ruthenium-labeled goat anti-human IgG F(ab')2 (1 μg/ml in DPBS at *pH* 6.0) for 1 h at room temperature. Cells were washed 3 times with DPBS at *p*H 6.0. Tris-based Read Buffer T without surfactant (MSD) was added to wells immediately before measuring Relative Luminscent Units (RLU) using a Sector Imager 6000 reader and Discovery Workbench software (MSD). For analysis, background signal RLUs were subtracted from RLUs of samples run in triplicate.

## Results

### Multiple sources of human intestinal epithelial cells exhibit intestinal marker expression

Along with limited viability in culture, one of the major drawbacks of routinely using primary hInEpCs for studying intestinal physiology is that these cells must be collected from human donors. A newly available commercial source of primary hInEpCs greatly increases the convenience of obtaining primary cell stocks, but need to be further characterized for intestinal cell-type specific marker expression and functional monolayer formation. Thus, we sought to characterize these commercially available stocks to assess their physiological relevance for use in cell-based *in vitro* assays of intestinal uptake and transport.

**Figure 2:**
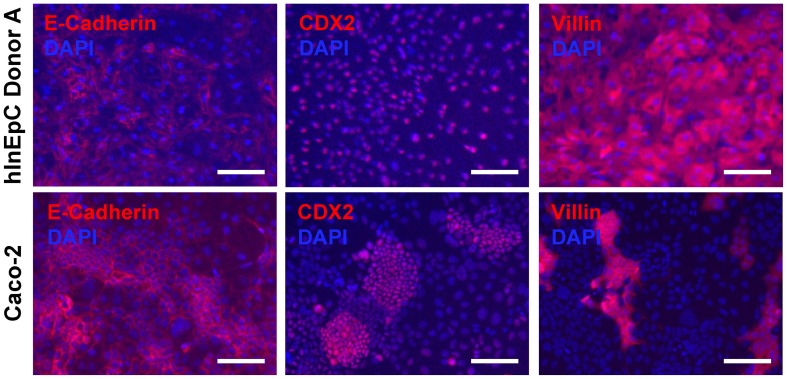
**Characterization of intestinal marker expression in commercially available (Lonza) primary human intestinal epithelial cells (hInEpCs) relative to immortalized human intestinal cell line Caco-2. Top row**: primary hInEpCs uniformly express intestinal epithelial markers E-Cadherin (tight junctions), CDX2 (hindgut), and Villin (enterocytes). Primary hInEpC Donor A shown (see Figure A1B for Donors B and C). **Bottom row**: Caco-2 show less uniform expression of enterocyte marker Villin. Scale bar, 100 μm.

As previously reported, a measure of intestinal cell phenotypic quality is the expression of a panel of general intestinal epithelial markers (Spence et al., 2010). We used immunocytochemistry to verify expression and expected localization of several representative intestinal epithelial markers within primary hInEpCs from three different donors (Figure 2, top row; Figure A1B), relative to the immortalized human intestinal epithelial cell line Caco-2 (Figure 2, bottom row). This was visualized for E-cadherin, an epithelial cell adhesion marker found within tight junctions between cells (Zbar et al., 2004), CDX2, a transcription factor that is upstream of signaling promoting intestinal cell fate (Gao et al., 2009) and Villin, an actin-binding protein associated with the intestinal brush border of absorptive enterocytes (Friederich et al., 1999). The localization of these markers within cells was appropriate in primary hInEpCs and Caco-2 (Figure 2). Marker expression intensity was variable between the three hInEpC donors within this study, with strongest expression evident in Donor A (Figure 2, top row; Figure A1B). Expression results for hInEpCs and Caco-2 were used to compare to iPSC-derived iHIOs grown in transwells (below).

**Figure 3:**
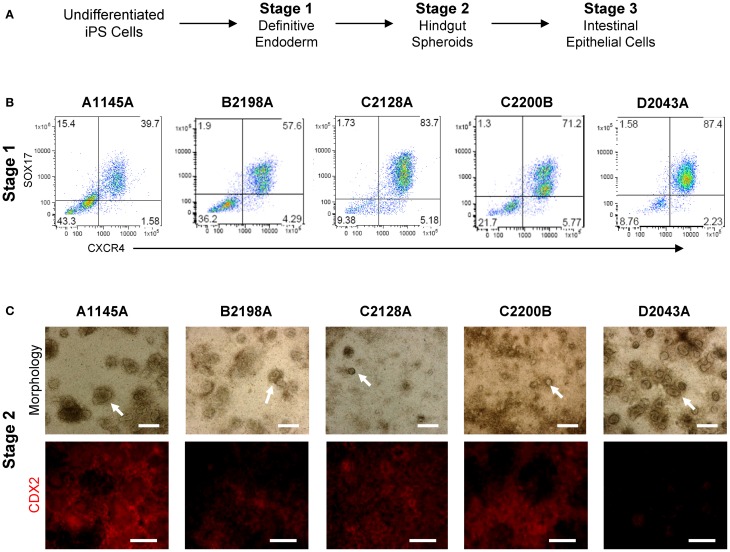
**iPSC directed intestinal differentiation in transwell culture results in cells with expected morphology and marker expression through Stage 2. (A)** Directed intestinal differentiation occurs through several distinct stages. **(B)** Transwell cells derived from a panel of iPSC lines express Definitive Endoderm markers SOX17 and CXCR4 at Stage 1. **(C)** Top row: transwell iPSC panel morphology at Stage 2 differs in amount and size of budding spheroids (arrow heads). Scale bar, 250 μm. Bottom row: CDX2 expression of Stage 2 transwell iPSC-derived cells differs among cell lines in intensity and uniformity. Scale bar, 250 μm.

In order to evaluate iPSC-derived intestinal cell phenotypes within classical *in vitro* monolayer conditions, we adapted intestinal organogenesis to transwell culture by performing iPSC differentiation through Stage 3 within transwell inserts (Table 2, Figure 3A). Differentiation of a panel of iPSC lines (A1145A, B2198A, C2128A, C2200B, and D2043A) on matrigel-coated transwell inserts using Myostatin, Glycogen Synthase Kinase 3β inhibitor and B27 supplement resulted in cells expressing definitive endoderm markers SOX17 and CXCR4 (Figure 3B), similar to iPSCs differentiated to Stage 1 in standard tissue culture plates (Kauffman et al., Submitted). Further differentiation of iPSC-derived definitive endoderm cells within transwell plates, by addition of Keratinocyte Growth Factor and Retinoic Acid, produced layers of cells with developing spheroid structures at Stage 2 in many of the iPSC lines (Figure 3C, top row). CDX2 expression at Stage 2 was relatively uniform within layers of cells differentiating in transwells (Figure 3C, bottom row). However, D2043A showed more intense expression around spheroid structures.

**Figure 4:**
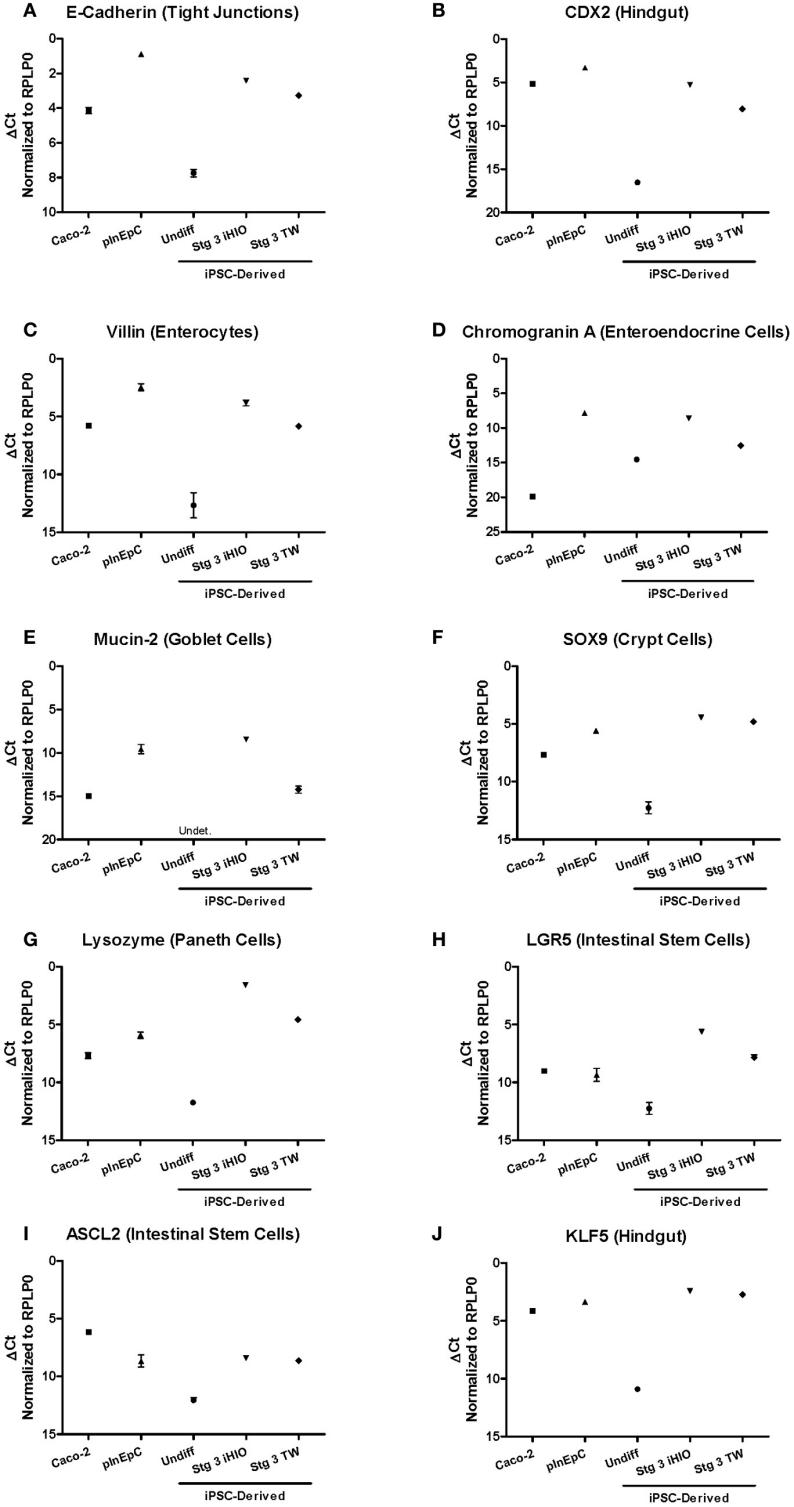
**Comparison of intestinal marker mRNA expression in multiple sources of human intestinal epithelial cells.** Expression of tight junction marker **(A)** and hindgut epithelia **(B)**, as well as specific epithelial lineage markers **(C–J)** intestinal markers of epithelial intestinal lineages was evaluated by RT-PCR in Caco-2, primary hInEpCs (average of Donors A–C), and iPSC-derived undifferentiated (Undiff), Stage 3 induced human intestinal organoids (Stg 3 iHIOs), or Stage 3 transwell intestinal cells (Stg 3 TW). Using the Δ Ct method, raw *Ct* values were normalized to housekeeping gene RPLP0. *N* = 3; Error bars represent SEM.

iPSC-derived Stage 3 3D intestinal organoids exhibit E-Cadherin, CDX2, Villin, and Chromogranin A immunoreactivity with the expected localization, (Kauffman et al., Submitted). In this study, a supply of transwell A1145A iPSC-derived Stage 3 Day 31 cells was limited, and did not allow sufficient material for extensive immunocytochemistry analyses of intestinal markers. Thus, mRNA expression analysis was used to directly compare larger panel of known intestinal marker and differentiation control genes Stage 3 A1145A iPSC-derived intestinal marker expression to Caco-2 and primary hInEpCs by RT-PCR (Figure 4). Differentiated Stage 3 iPSCs showed increased marker expression relative to undifferentiated cells, usually reaching a level more similar to primary hInEpCs in the case of 3D iHIOs (Figure 4), or more similar to Caco-2 in the case of transwell-differentiated iPSCs (Figure 4). Intestinal markers that followed this expression pattern included epithelial tight junction marker E-Cadherin (Zbar et al., 2004), hindgut epithelial marker CDX2 (Gao et al., 2009), enterocyte maker Villin (Friederich et al., 1999), enteroendocrine marker Chromogranin A (O'Connor et al., 1983), and Mucin-2 (MUC2), a marker for intestinal goblet cells (Gum et al., 1999) (Figures 4A–E).

**Figure A3:**
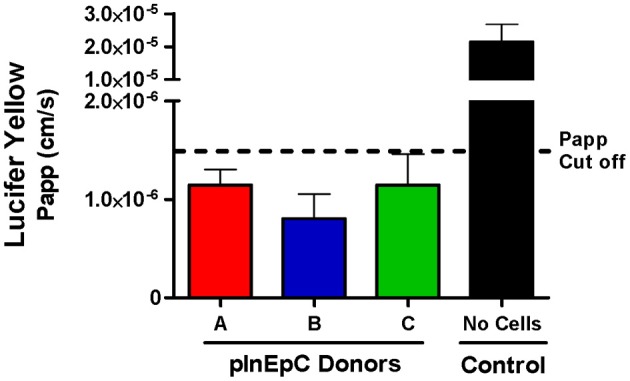
**All primary hInEpC donors used within the P-glycoprotein transport assay showed an average lucifer yellow apparent permeability (Papp) below accepted cutoff for intact monolayers 1.5 × 10^-6^ cm/s.** Only transwells that met this cutoff were used to calculate Papp ratios for Digoxin and Atenolol (Figure 6). *N* = 3; Error bars represent SEM.

For some intestinal makers, expression in iPSC-derived intestinal cell types was highest in iPSC-derived cells. For example, expression levels of intestinal crypt cell marker Sex determining region Y-box 9 (SOX9), paneth cell marker Lyzosyme (LYZ) (Peeters and Vantrappen, 1975), and intestinal stem cell marker LGR5 (Barker et al., 2007) were greatest in iHIOs and transwell differentiated cells compared to Caco-2 or primary hInEpCs (Figures 4F–H). On the other hand, an additional intestinal stem cell marker, Achaete Scute-Like 2 (ASCL2) (van der Flier et al., 2009), was most highly expressed in Caco-2 (Figure 4I). In the case of intestinal epithelial cell differentiation transcription factor Kruppel-like factor 5 (KLF5) (Bell et al., 2013), mRNA expression levels were similar for all intestinal cell types (Figure 4J). The expression of several control genes [e.g., pluripotent marker Octamer-binding Transcription factor 4 (OCT4) (Nichols et al., 1998)] confirmed the cell sample quality (Figure A3).

### Primary hInEpCs and iPSC-derived intestinal cells form transwell monolayers with tight junctions

**Figure 5:**
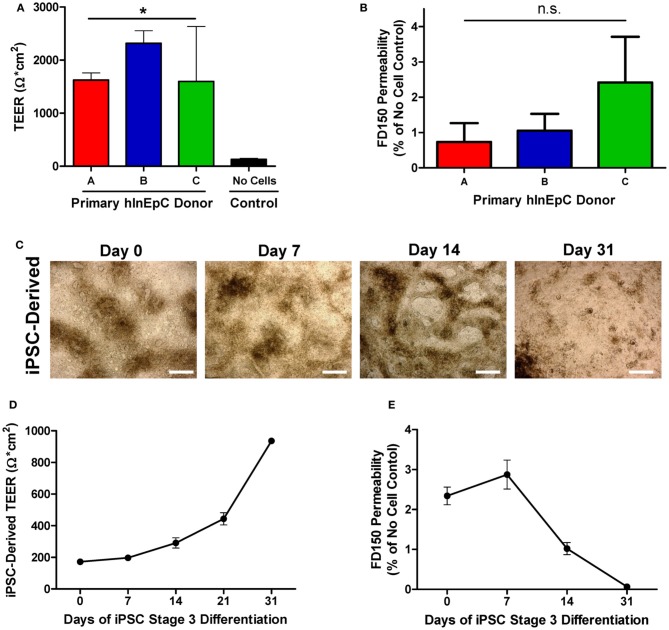
**Primary hInEpCs and iPSC-derived cells develop transwell monolayers with comparable integrity. (A)** Primary hInEpC monolayers exhibited Transepithelial Electrical Resistance measurements of > 1500 Ω × cm^2^. **(B)** All primary hInEpC donors exhibit FITC-Dextran, molecular weight 150 kDa (FD150) permeability below 3%, relative to no cell control. **(C)** A1145A-derived cells lose dense structures during transwell differentiation in Stage 3 media, leaving behind confluent monolayer-like cells by Day 31. Scale bar, 250 μm. **(D)** Differentiating iPSCs (cell line A1145A shown) are capable of exhibiting increasing TEER during Stage 3 differentiation, reaching > 900 Ω × cm^2^ by Day 31. **(E)** A1145A cells exhibit low FD150 permeability with increasing days in Stage 3 culture relative to no-cell control. *N* ≥ 3; Error bars represent SEM. For panels **(A)** and **(B)**, significance was determined by One-Way ANOVA, ^*^p < 0.01.

While all three primary hInEpCs donors appeared to be capable of forming confluent cell layers in transwell culture that expression the tight-junction marker E-Cadherin (Figures 2, 4A), we assessed tight junction formation functionally through measurements of Transepithelial Electrical Resistance (TEER) and monolayer permeability. As confluent epithelial monolayers form, TEER measurements generally increase, reaching 260–420 Ω × cm^2^ on average in Caco-2 cultures (Le Ferrec et al., 2001). We found that after 11 days of transwell culture, primary hInEpCs from all three donors exhibited TEER measurements of > 1500 Ω × cm^2^ (Figure 5A), providing a strong indication of tight junction formation. As changes in epithelial TEER can also be explained by changes in transcellular ion permeability (Yu and Sinko, 1997), we confirmed tight junction formation of pInEpC transwell cultures by determining the monolayer permeability to FITC-labeled Dextran at a molecular weight of 150,000 (FD150). Apical side incubation in transwell chambers, resulted in <3% FITC-labeled Dextran detected on the basolateral side, relative to control transwells with no cells for primary hInEpC Donors A and B (Figure 5B). Donor C, which corresponds to the cells with highly variable TEER measurements (Figure 5B) showed slightly more FITC-Dextran permeability (~3% relative to control transwells).

While Stage 3 Day 31 transwell A1145A iPSC-derived cells exhibited intestinal marker expression consistent with other intestinal epithelial cell sources, it was difficult to gauge at which point during Stage 3 differentiation iPSC-derived cells may begin to take on functional phenotypes. Thus, we performed a time course experiment in which A1145A iPSCs were differentiated within transwell culture and assessed for monolayer morphology and evidence of tight junction formation throughout Stage 3 (Days 0, 7, 14, 21, 31). Morphologically, A1145A iPSC-derived cells showed 3D structures and dense patches of cells within transwells at the beginning of Stage 3 that appeared to disappear as differentiation progressed, leaving a flat monolayer-like layer of cells by Day 31 of Stage 3 (Figure 5C). To assess tight junction formation in confluent A1145A iPSC-derived transwell monolayers, we measured TEER and FITC-Dextran permeability during iPSC transwell differentiation. At Day 0 of Stage 3 differentiation, A1145A iPSC-derived cells within transwells exhibited low TEER measurements of less than 200 Ω ×cm^2^ which steadily increased to measurements reaching 937 Ω ×cm^2^ by Day 31 (Figure 5D). At Stage 3 Day 0, iPSC-derived cells showed FD150 permeability of ~2% relative to the no cell control (Figure 5E), a range similar to that seen for primary hInEpCs (Figure 5B). Coinciding with changes in TEER measurements during differentiation (Figure 5D), FD150 permeability decreased for iPSC-derived cells to as low as 0.06% of the no cell control (Figure 5E).

### Initial assessment of intestinal epithelial transport function

**Figure 6:**
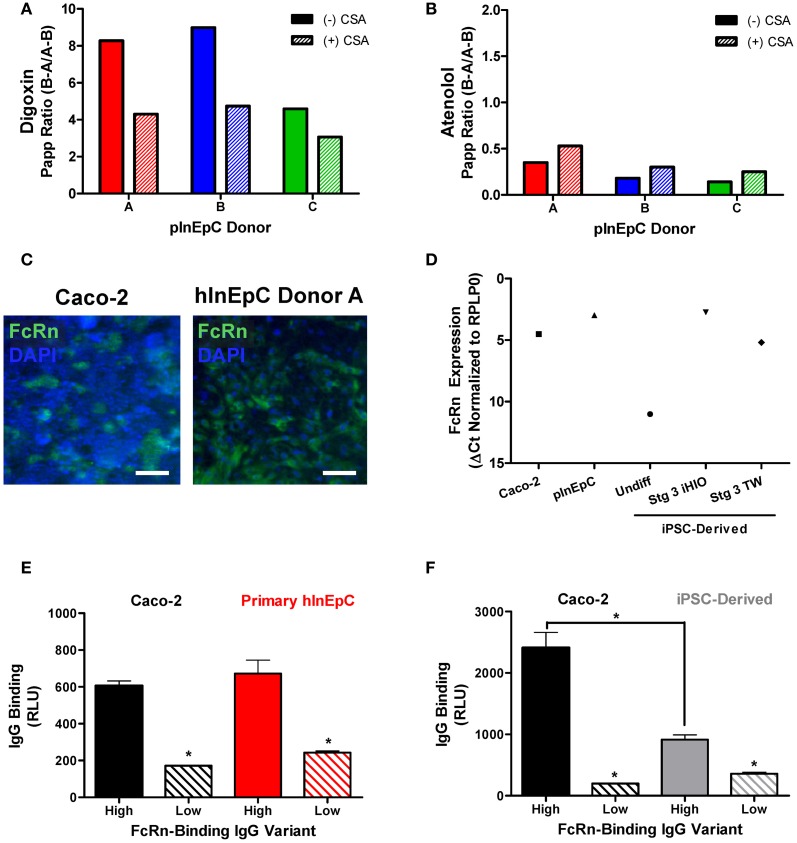
**Primary hInEpCs and iPSC-derived intestinal monolayers show functional activity in transwell culture. (A)** pInEpC donors A and B show Digoxin Papp Ratios >8, which are decreased in the presence of cyclosporin A (CSA), suggesting that these cell lines have P-glycoprotein (Pgp) transport activity. **(B)** Atenolol Papp ratios for all samples fell below the expected <2 cutoff for Pgp activity. **(C)** Primary hInEpCs (right) show more uniform intracellular FcRn expression than Caco-2 (left), as shown by immunofluorescence. Scale bar, 100 μm. **(D)** iPSC-derived transwell cells express FcRn mRNA levels similar to Caco-2, but lower than primary hInEpCs and iPSC-derived 3D induced human intestinal organoids (iHIOs). Caco-2 **(E,F)**, primary hInEpCs **(E)** and iPSC-derived transwell cells **(F)** show FcRn-dependent mAb binding by MSD assay, as demonstrated by statistically greater cell binding of high FcRn-binding IgG variants [High, N434A **(E)** or M428L **(F)**] than low FcRn-binding variants (Low, H435A). *N* ≥ 4; Error bars represent SEM. **(E,F)**, statistical significance determined by *t*-test, ^*^p < 0.001.

To further validate these cell sources functionally, we assessed monolayers for molecule transport or binding. A previously established mechanism influencing small molecule transport by intestinal epithelial cells is efflux by membrane associated ATP-binding cassette P-glycoprotein (Pgp) transporters, which facilitate cellular efflux to prevent accumulation of their substrates (Murakami and Takano, 2008). To assess Pgp transport activity for a given substrate, the basolateral to apical (B–A) permeability is compared to apical to basolateral (A–B), where compounds with efflux ratios (B–A/A–B) greater than 2 or 3 are generally considered to be Pgp substrates (Balimane et al., 2006). Using transwell monolayers of primary hInEpCs, we found that efflux ratios for Digoxin, a compound known to be highly effluxed by Pgp (Balimane et al., 2006), were >8 for pInEpC Donors A and B (Figure 6A). Importantly, efflux of Digoxin was reduced when these cells were also treated with CSA, a known Pgp transporter inhibitor (Watanabe et al., 1997). Atenolol, a poorly Pgp-effluxed compound (Balimane et al., 2006) showed very low efflux ratios of <1 in all three primary hInEpC donors, which was not further reduced by CSA (Figure 6B). During the Pgp transport assay, transwells were also dosed apically with lucifer yellow to confirm primary hInEpC monolayer integrity based on permeability of this fluorescent molecule (Figure A3). While the average apparent permeability for all three primary hInEpC donors was below 1.5 × 10^−6^ cm/s, only transwells below this standard cutoff were used for analysis of Digoxin and Atenolol flux.

Unfortunately, as A1145A iPSC-derived transwell intestinal cells were very limited, there were insufficient cells within this study to perform a properly-controlled Pgp transport assay. Thus, to further validate iPSC-derived cells, we assessed FcRn immunoreactivity and performed a cell surface binding assay for neonatal Fc receptor (FcRn)-mediated transport, which was amenable to the limited cell supply.

**Figure A4:**
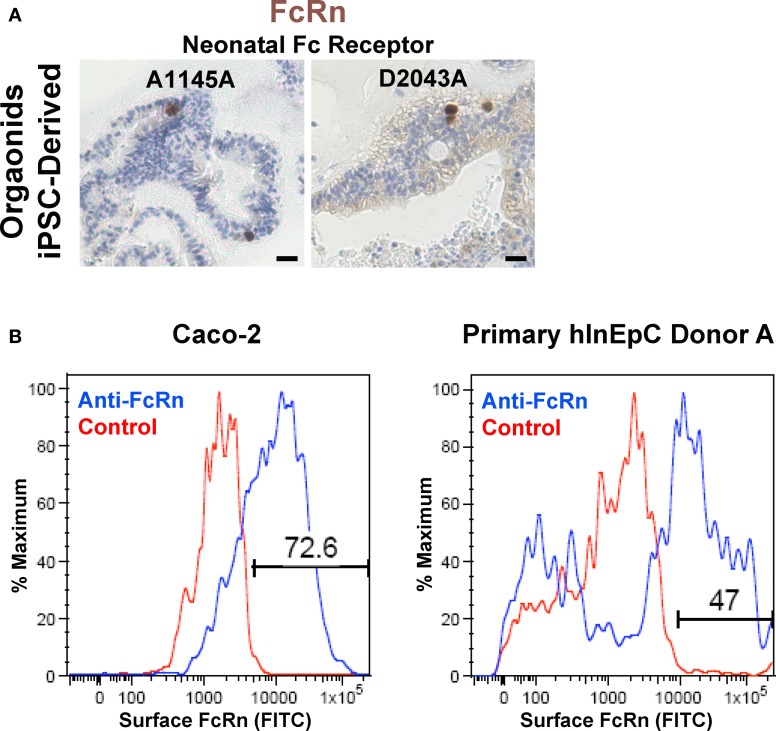
**Intestinal epithelial cell expression of FcRn. (A)** Immunohistochemical analysis demonstrates iPSC-derived Stage 3 intestinal organoid expression and punctate localization of neonatal Fc Receptor expression in iPSC-derived organoids. Scale bar, 100 μm. **(B)** Flow cytometry analysis shows surface FcRn expression on Caco-2 (left) and primary hInEpCs (right, Donor A shown).

Intense FcRn expression in iPSC-derived intestinal organoids was noted as intense single-cell expression within the population of cells (Figure A4A) similar that previously reported for human intestinal tissue (Dickinson et al., 1999). We confirmed FcRn expression immunocytochemically in transwell monolayer cultures of Caco-2 and primary hInEpCs both intracellularly (Figure 6C) and on the surface of cells (Figure A4B). Real-Time RT-PCR analysis showed that FcRn mRNA expression increases to the level close to that of Caco-2 by Stage 3 of transwell differentiation (Figure 6D), whereas FcRn expression was highest in primary hInEpCs and iHIOs.

We examined binding of high or low FcRn-binding IgG mAb variants to Caco-2, primary hInEpCs, or iPSC-derived transwell intestinal cells by Meso Scale Discovery assay. Use of this highly sensitive ELISA-like assay allowed us to perform FcRn-dependent binding experiments with proper controls, even with limited-supply iPSC-derived cells. Similar to Caco-2, primary hInEpCs showed significantly higher binding of the IgG variant with a high affinity to FcRn (N434A) than the low FcRn-binding variant (H435A) (Figure 6E). iPSC-derived intestinal cells also demonstrated significantly higher binding of a high FcRn-binding IgG variant (M428L) relative to the low FcRn-binding variant (H435A), however, maximal cell binding was not as high as Caco-2 (Figure 6F).

## Discussion

The major findings of this study are that marker expression of primary hInEpCs and iPSC-derived intestinal cells were on the same order of magnitude, or better than Caco-2, as determined by immunocytochemistry and mRNA expression analyses. The iPSC-derived intestinal cells were successfully adapted to differentiation within 2D transwell monolayer culture, and similar to primary hInEpCs, demonstrated functional tight junctions with TEER and low permeability similar to, or better than, Caco-2. Initial assessment suggested functional activity for intestinal transporters such as Pgp transport (primary hInEpCs) or FcRn-dependent binding of molecules (primary hInEpC and iPSC-derived cells). The main conclusions from our study are further discussed below in relation to previously published studies.

Before using a recently released commercially available source of primary hInEpCs for comparing iPSC-derived intestinal cells, it was necessary to more fully characterize this intestinal epithelial cell source for marker expression and barrier function. The extent to which primary hInEpCs expressed markers by immunofluorescence varied, with expression of E-Cadherin, Villin, and CDX2 strongest in Donor A; however, intestinal marker expression appeared to be more consistent between donors by RT-PCR analysis. While all three donors exhibited high TEER (>1500 Ω × cm^2^), TEER and FD150 permeability showed high variability in Donor C. Moreover, Donor C showed poor efflux of Pgp-transported Digoxin, suggesting that this primary hInEpC donor is less useful for intestinal barrier function and transport than Donors A and B.

A 3D directed intestinal organogenesis protocol was successfully adapted to 2D transwell differentiation, based on intestinal marker similarity. Stage 1/Definitive endoderm markers SOX17 and CXCR4 were expressed in 40–87% of the cell population. Stage 2-marker CDX2 was also robustly expressed within transwell cultures. Stage 3 differentiation within transwells of iPSC-line A1145A (Kauffman et al., Submitted) was similar to the development of iHIOs, except that spheroid structures that formed by Stage 2 of differentiation were not excised for 3D culture, but allowed to continue differentiation within the adherent layer of cells. Interestingly, spheroid structures found at Stage 2 were gradually lost, so that by Stage 3, Day 31, a monolayer-like morphology was found within transwell cultures, which showed TEER measurements more similar to primary hInEpCs and greater than the average TEER for Caco-2 transwell cultures (Le Ferrec et al., 2001). Consistent with this result, low permeability of FD150 for primary hInEpCs and iPSC-derived transwell cells suggests that a barrier function was present in these cell cultures. It is important to note that the TEER obtained in this study for human intestinal epithelial cell sources is several-fold higher than typically found in intestinal tissue *ex vivo*; however, high TEER values may not discount the physiological relevance of these cells *in vitro*, as the presence of several epithelial layers and cell types within intestinal tissue can add multiple impedances to intestinal transmural TEER measurements (Gitter et al., 1998, 2000).

**Figure A5:**
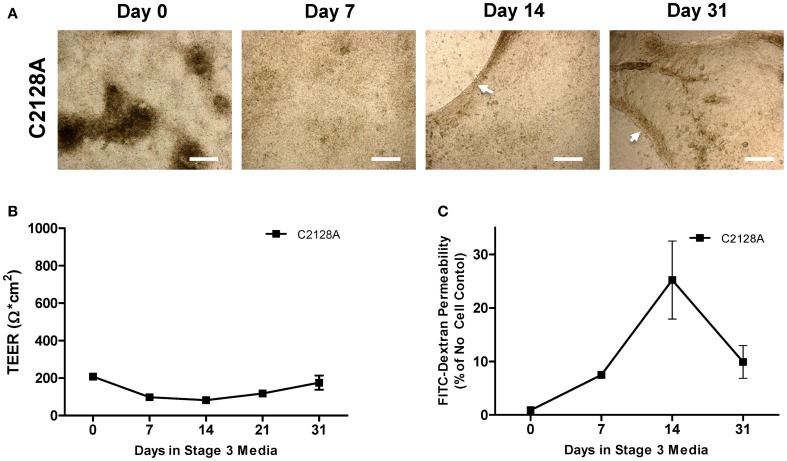
**Not all iPSC lines are capable of producing transwell intestinal cell monolayers with characteristics suggestive of tight junction formation. (A)** Morphology of during C2128A transwell differentiation shows that cells lose dense structures during differentiation; however, the monolayer begins to pull into itself during late Stage 3 differentiation (arrow heads), resulting in large holes on transwell inserts. Scale bar, 250 μm. **(B)** C2128A cells do not show increasing TEER with increasing days in Stage 3 culture. **(C)** C2128A shows high FD150 permeability at later stages of differentiation. *N* = 3; Error bars represent SEM.

In addition to A1145A, which shows consistent robust intestinal differentiation in 3D and 2D monolayer culture, we also performed a transwell differentiation time course on C2128A because this cell line showed uniform expression of CDX2 at Stage 2 within transwell culture, but demonstrated a distinct morphology. At Stage 2, C2128A cells appeared to already exhibit a monolayer-like morphology with little to no dense structures; therefore, we reasoned that C2128A might be more amenable to the development of iPSC-derived intestinal cells for use in functional transwell monolayer assays. However, as Stage 3 differentiation of C2128A-derived progressed, holes appeared within the layer of cells, which corresponded to low TEER measurements and high FD150 permeability at Stage 3 (Figure A5). Thus, differences in iPSC line intestinal differentiation capacity appear to be evident during 2D transwell culture differentiation.

mRNA expression analysis of an extensive panel of intestinal marker and control genes enabled a direct comparison of primary hInEpCs and iPSC-derived cell maker expression to Caco-2 cells. As expected, undifferentiated iPSCs showed levels of marker expression several fold lower than all intestinal samples with the exception of the pluripotent cell control marker OCT4 (Nichols et al., 1998). Differentiated iPSC-derived iHIOs and transwell cells exhibited upregulated intestinal marker expression that was more similar to primary hInEpCs than Caco-2, consistent with a better *in vitro* representation of human intestinal epithelia than Caco-2.

As might be expected, expression of definitive endoderm marker CXCR4 was highest in Stage 3 iHIOs and transwell cells, which went through a definitive endoderm intermediate at Stage 1. Similarly, expression of the mesenchymal marker Vimentin was also most highly upregulated in iPSC-derived intestinal cells, relative to Caco-2. This result is consistent with previously reported increases in expression of Vimentin and another mesenchymal marker, Forkhead Box F1 (Mahlapuu et al., 1998), in intestinal cells derived from human embryonic stem cells, any may indicate the development of intestinal subepithelial myofibroblasts (Spence et al., 2010).

The intestinal stem cell marker LGR-5 (Barker et al., 2007) was highly upregulated in iPSC-derived intestinal cells relative to Caco-2, which may reflect more active intestinal cell proliferation within these cells, particularly in iHIOs. Expression of Paired Box Gene 6 (PAX6), with known roles in brain development (Mastick et al., 1997), was also highly expressed in iHIOs cells, with less expression in Stage 3 iPSC-derived transwell cells or the other intestinal sources tested. This is consistent with the high Chromogranin A expression in the development of a more mature enteroendocrine system within these cells, as PAX6 also has a known role in the development of Glucagon-like peptide (GLP)-1 and GLP-2 secreting cells (Fujita et al., 2008; Ye and Kaestner, 2009).

While intestinal marker expression in primary of iPSC-derived cells was very encouraging, it was unclear how functional these cells are compared to other established *in vitro* intestinal models, such as Caco-2 or primary tissue. For example, in pluripotent stem-cell derived models of liver or pancreas, progenitor cells must be engrafted into whole animals to produce mature, fully-functional cell types (Liu et al., 2011; Rezania et al., 2011). We realized that adaptation of directed intestinal organogenesis to 2D transwell culture would allow a more direct comparison of iPSC-derived intestinal cell barrier and transport functions to previously characterized intestinal epithelial cell models *in vitro*. Well-studied intestinal transport mechanisms within Caco-2 and primary human intestinal tissue include efflux of small molecules by membrane associated Pgp transporters, (Murakami and Takano, 2008), and intestinal receptor-mediated antibody transcytosis by FcRn (Dickinson et al., 1999; Claypool et al., 2004). These mechanisms were used as an initial assessment of functional quality of primary hInEpCs and iPSC-derived intestinal cells.

Primary hInEpCs A and B showed cyclosporine A-dependent efflux of Digoxin, but not Atenolol, indicative of the presence of Pgp transport activity in these cells. In addition to primary hInEpCs (Donor A), Stage 3 iPSC-derived transwell cells showed FcRn expression and FcRn-dependent mAb binding using high or low FcRn-binding IgG variants; however, unlike primary hInEpCs, iPSC-derived IgG binding did not occur to as large an extent as Caco-2. It should also be noted that in all FcRn-binding experiments, the cell binding signal, while well above background noise, was relatively low for this ELISA-based assay. Furthermore, since FcRn is expressed in neonatal and adult intestinal epithelial cells in primates (Israel et al., 1997; Dickinson et al., 1999) and marker expression during differentiation of iHIOs closely resembles that of embryonic intestinal development (Spence et al., 2010), it is still unclear whether the phenotype of these cells more closely resembles embryonic or adult intestinal tissue.

In summary, our studies indicate that iPSC-derived intestinal cells and newly commercially available primary hInEpCs may provide an alternative source of physiologically relevant hInEpCs. Future studies will be needed to further evaluate the function of primary hInEpCs and iPSC-derived intestinal cells. Additionally, as these cells are still limited by their viability (hInEpCs) and extensive differentiation time (iPSC-derived cells), large-scale studies will require additional development of methods for scale up or long-term maintenance of these transwell cultures, such as non-oncogenic immortalization strategies.

### Conflict of interest statement

All investigators are employees of Johnson & Johnson and have no other declarations of interest to disclose.

## Figures and Tables

**Figure A2:**
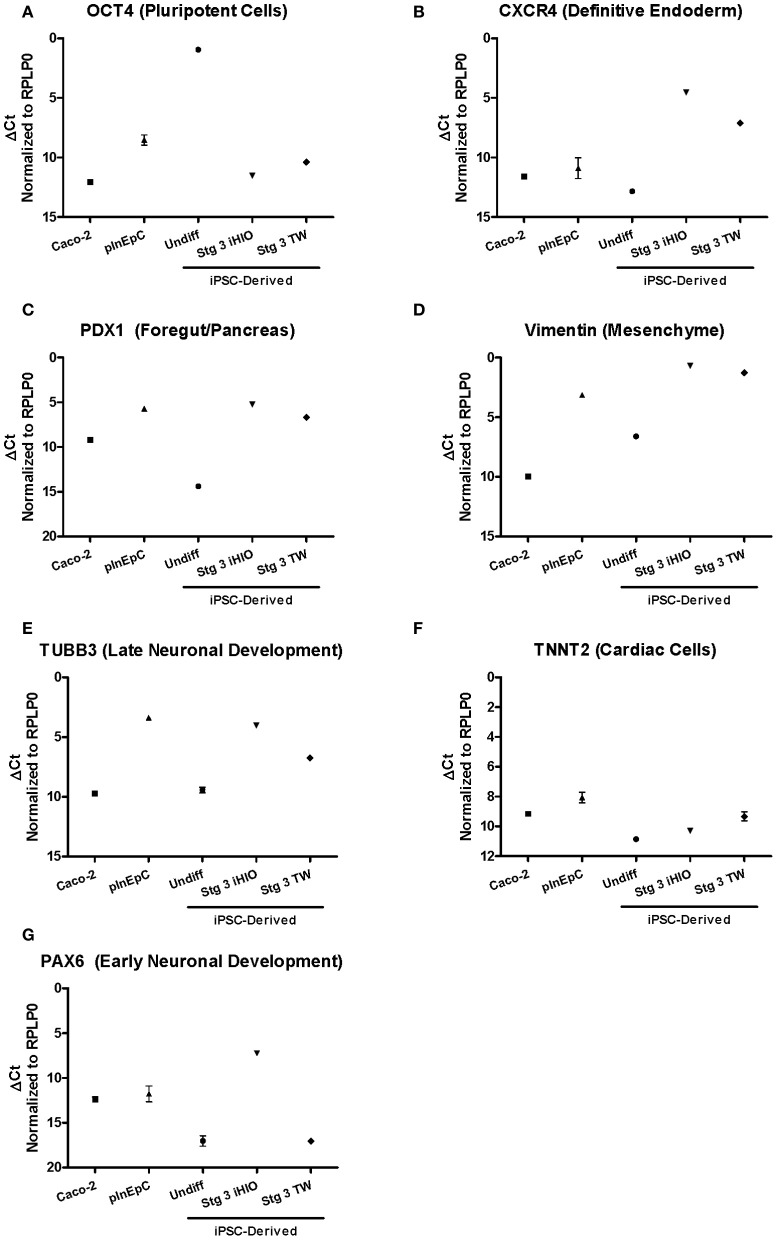
**Validation of intestinal cell RT-PCR analysis through a panel of control/non-intestinal markers.** Expression of intestinal markers was evaluated by RT-PCR in Caco-2, primary hInEpCs (average of Donors A–C), and iPSC-derived undifferentiated (Undiff), Stage 3 induced human intestinal organoids (Stg 3 iHIOs), or Stage 3 transwell intestinal cells (Stg 3 TW). Using the DCt method, raw *Ct* values were normalized to housekeeping gene RPLP0. *N* = 3; Error bars represent SEM.

## Appendix

### Results

In addition to intestinal cell-specfic markers (Figure 4), other control markers were assessed by RT-PCR to evaluate sample quality of iPSC-derived cells, Caco-2, and primary intestinal cells (Figure A2). iPSC-derived cells and primary intestinal cells showed very low expression of pluripotent cell transcription factor POU domain, class 5 (OCT4) (Nichols et al., 1998), and about 2000-fold lower than undifferentiated cells (Figure A2A). To determine how successfully iPSC-derived intestinal cells differentiated away from a definitive endoderm cell type after Stage 1, expression of CXCR4 was analyzed. As somewhat expected, expression of this definitive endoderm marker was highest in iPSC-derived cells, which went through a definitive endoderm intermediate at Stage 1, with expression levels ranging about 150-fold higher relative to Caco-2 (Figure A2B). Expression of the pancreatic foregut marker PDX1 (Miller et al., 1994) was found to be 10–15-fold higher in iPSC-derived cells and primary hInEpCs relative to Caco-2 (Figure A2C). Similarly, expression of the mesenchymal marker Vimentin was also highly upregulated in primary hInEpCs and iPSC-derived intestinal cells, relative to Caco-2 (Figure A2D).

Neuronal marker Tubulin, beta 3 (TUBB3) (Poirier et al., 2010), expression was higher in iPSC-derived cells and primary hInEpCs relative to Caco-2 (Figure A2E). Expression of cardiac cell marker Troponin T, type 2 (TNNT2) (Townsend et al., 1994) was the comparable across intestinal cell types (Figure A2F). Expression of Paired Box Gene 6 (PAX6), with known roles in brain development (Mastick et al., 1997), was highly expressed only Stage 3 iHIOs (Figure A2G).
